# Imaging Collagen in Scar Tissue: Developments in Second Harmonic Generation Microscopy for Biomedical Applications

**DOI:** 10.3390/ijms18081772

**Published:** 2017-08-15

**Authors:** Leila Mostaço-Guidolin, Nicole L. Rosin, Tillie-Louise Hackett

**Affiliations:** 1 Centre for Heart Lung Innovation, University of British Columbia, Vancouver, BC V6Z 1Y6, Canada; Leila.Mostaco-Guidolin@hli.ubc.ca (L.M.-G.); Nicole.Rosin@hli.ubc.ca (N.L.R.); 2 Department of Anesthesiology, Pharmacology and Therapeutics, University of British Columbia, Vancouver, BC V6Z 1Y6, Canada

**Keywords:** second harmonic generation, nonlinear optical microscopy, scar tissue, fibrillar collagen, skin, lung, vessels, image analysis

## Abstract

The ability to respond to injury with tissue repair is a fundamental property of all multicellular organisms. The extracellular matrix (ECM), composed of fibrillar collagens as well as a number of other components is dis-regulated during repair in many organs. In many tissues, scaring results when the balance is lost between ECM synthesis and degradation. Investigating what disrupts this balance and what effect this can have on tissue function remains an active area of research. Recent advances in the imaging of fibrillar collagen using second harmonic generation (SHG) imaging have proven useful in enhancing our understanding of the supramolecular changes that occur during scar formation and disease progression. Here, we review the physical properties of SHG, and the current nonlinear optical microscopy imaging (NLOM) systems that are used for SHG imaging. We provide an extensive review of studies that have used SHG in skin, lung, cardiovascular, tendon and ligaments, and eye tissue to understand alterations in fibrillar collagens in scar tissue. Lastly, we review the current methods of image analysis that are used to extract important information about the role of fibrillar collagens in scar formation.

## 1. Introduction

The ability to respond to injury with tissue repair is a fundamental property of all multicellular organisms. In the seconds after injury, various intracellular and intercellular pathways must be activated and synchronized to respond if tissue integrity and homeostasis are to be restored. In general, the wound repair process in almost all tissues involves activation of the cellular components of the immune system (neutrophils, monocytes, lymphocytes and dendritic cells), the blood coagulation cascade and the resultant activated inflammatory pathways. In addition to immune cells, multiple structural cell types are activated (endothelial cells, epithelium, keratinocytes, and fibroblasts), which undergo marked changes in phenotype and gene expression leading to cell proliferation, differentiation, migration and extracellular matrix production [1,2]. Once the wound repair response has successfully regained tissue homeostasis, it must be controlled and switched off. Interestingly, malignant transformation is an uncommon event in repairing wounds [3,4]. The human fetus and some adult eukaryotic organisms can respond to injury through regeneration leading to restoration of the original tissue architecture [5]. In adult humans, this ability is lost through unknown processes, and wound repair normally results in non-functional tissues formed by a patch of cells (mainly fibroblasts) and disorganized extracellular matrix (mainly fibrillar collagens) that is commonly referred to as scar tissue.

Demographically, the number of individuals suffering from chronic wounds and impaired healing conditions continues to rise leading to health and economic burdens to society [6,7,8,9]. As an example, defective wound repair and chronic wounds can result from myocardial scar tissue leading to congestive heart failure after myocardial infarction, cirrhosis of the liver and lung fibrosis in response to toxin-mediated injury, or hypertrophic scars to surgical wounds. Understanding the underlying molecular basis of tissue repair and its failure is therefore an important unmet clinical need.

Studying wound repair in various conditions and models will expand our understanding of the wound repair process in humans that will hopefully lead to the identification of novel pathways or molecular signals that can be therapeutically targeted to restore their lost regenerative capacity. Over the last 15 years, nonlinear optical microscopy (NLOM) has emerged as a powerful research tool [10,11,12,13] for visualizing the supramolecular assembly of collagen in tissues at an unprecedented level of detail [14,15,16,17]. Based on the physics of nonlinear light-matter interactions, and using these interactions as contrast mechanisms for cellular and tissue imaging investigations, modalities such as Second Harmonic Generation (SHG), Third Harmonic Generation (THG), Coherent anti-Stokes Raman (CARS), and two-photon excitation fluorescence (TPEF) have acquired a reputation as an excellent optical tool for answering multiple biological questions [13,18,19]. The development of NLOM imaging has enhanced basic biomedical research, and provided a new suite of quantitative metrics for the diagnosis of a wide range of diseases [20,21,22,23,24,25,26,27,28,29,30,31,32,33,34,35,36,37,38].

In this review, we describe the physical characteristic of SHG, and the current NLOM systems that are used by biologists for SHG imaging. We provide an extensive review of studies that have used SHG in wounding and repair of skin, lung, cardiovascular, tendon and ligament, and eye tissues to understand alterations in fibrillar collagens in scar tissue [39,40,41,42]. It is important to note that SHG imaging is used to study many other diseases, including cancer, but this review is limited to the tissues listed above. Lastly, we provide an overview of the current methods of image analysis that are used to extract important information about the role of fibrillar collagens in scar formation.

## 2. The Extracellular Matrix

The extracellular matrix (ECM) is essential for the normal development, function and homeostasis of all eukaryotic cells [43,44,45,46]. While providing the physical matrix that separates establishing and established tissues and organs, the ECM also actively participates in regulating the abundance of growth factors, receptors, level of hydration and pH of the local tissue environment. The exquisite tissue specific functions of the ECM are achieved through its complex biochemical composition (water, proteins and polysaccharides) and dynamic biophysical properties [43,44,45,46]. ECM molecules are generally formed from small, modular repeating subunits that form homo or heteropolymers that become supramolecule assemblies with highly specialized functions. In all cases, each class of ECM molecule has evolved the ability to interact with other classes of ECM molecules to produce unique structural and biochemical properties. Therefore ECM molecules can function as support for cells (complex adhesion surfaces, diffusion barriers) or act as active participants in cell signaling (binding domains for growth factors and chemokines) [47,48,49,50]. The ECM is primarily composed of two classes of macromolecules: glycoproteins (such as fibronectin, proteoglycans and laminin) and fibrous proteins (including collagens and elastin). This review focuses on the structural properties of fibrillar collagens and how the NLOM technique SHG can be applied to understand their biophysical properties in organs during health and disease.

## 3. Fibrillar Collagen

Collagens form a heterogeneous family of fibrous proteins of which there are 28 different types identified in vertebrates. In animals, the collagen family represents the most abundant protein [51] and collagen is the dominant protein within the ECM. Here, we focus on the fibrillar collagens, which are capable of withstanding tensile forces within the ECM and have a non-centrosymetric structure that can be imaged using NLOM.

At the structural level, all collagen molecules are made up of three polypeptide α chains that form homo- or heterotrimers. The prototypical α chain in all fibrillar collagens consists of approximately 338 repeating Gly-X-Y- triplets called a triple-helical motif (where X and Y can be any amino acid but are frequently proline and hydroxyproline) that is flanked by two non-collagenous domains, the N- and C-propeptides [52,53,54,55]. Once transcribed and within the endoplasmic reticulum, the triple-helix structure results in the intertwining of the three α chains starting at the C-terminal propeptide, forming a right-handed superhelix that is further stabilized by hydroxylation of particular lysine and proline residues (O-linked glycosylation). This assembly results in a rod-like structure 300 nm in length and 1.5 nm in diameter, termed procollagen, which is packaged for export by the Golgi apparatus. Procollagen is then converted into mature collagen by the removal of N- and C-propeptides via collagen type-specific metalloproteinase enzymes, within plasma membrane extrusions (known as fibripositors) that project from the cell surface. Mature collagen molecules then have the ability to engage in self-association to form microfibrils at the cell surface that can merge and grow both longitudinally and axially. To form mature collagen fibres, lysyl oxidases covalently crosslink lysine residues within the supramolecular assembly, providing stability and mechanical properties [45,56,57,58,59,60]. Conversely, non-fibrillar collagens contain non-triple helix regions, which lead to kinks in the resulting macromolecular structure that straighten under small strains. This means that non-fibrillar collagens do not form centrosymetric structures and cannot be visualized by SHG.

## 4. Collagen Imaging Using Second Harmonic Generation (SHG) Microscopy

SHG microscopy has emerged as a useful tool for studying key facets of collagen remodeling. SHG imaging is an attractive alternative to conventional or fluorescent-based histology for studying tissue composition and visualizing the molecular structure of collagen due to its label-free nature, high sensitivity and specificity [14,19,61,62,63]. The optical sectioning capability of SHG also provides a means of imaging bulk tissue in 3 dimensions (3D).

### 4.1. What Is SHG and How Does It Work?

**Figure 1:**
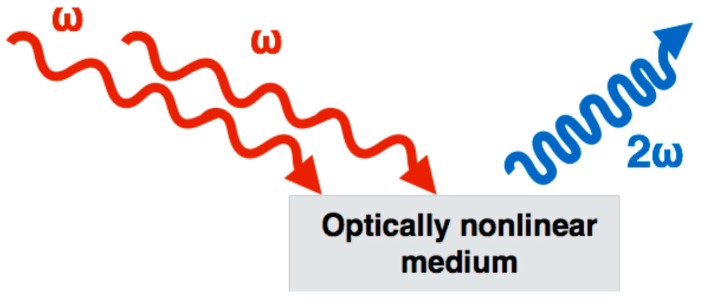
Second-harmonic generation (SHG) is a nonlinear optical process, in which two photons interacting within a nonlinear material are effectively “combined” to form a new photon with twice the energy (2ω), and therefore twice the frequency, or half the wavelength of the initial photons.

In short, SHG is a process that occurs when two photons are combined in an optically nonlinear medium, lacking in centro-symmetry (such as collagen), creating a SHG photon with a wavelength exactly half of the excitation wavelength (or twice the frequency, ω), as illustrated in Figure 1.

Before we begin explaining SHG, we need to first take a step back and define what light is. Light is part of the electromagnetic spectrum, and electromagnetic radiation waves are defined as fluctuations of electric and magnetic fields, which can transport energy between locations. Light, or electromagnetic radiation, can also be described as a stream of photons, mass-less particles that travel at the speed of light with wavelike properties.

The optical response of a material is expressed in terms of the induced polarization. Polarization describes the response of both the material and the applied electric field to one another. Polarization can be used to calculate the forces that result from these interactions [64,65,66]. For a linear material, the relationship between the polarization **P** and the electric field **E** of the incident radiation is linear:

**P** = ε_0_·χ^(1)^**E** (1)

where χ^(1)^ is the linear susceptibility (a dimensionless proportionality constant). The linear susceptibility due to an applied electric field indicates the degree of polarization of a dielectric material. A material with a higher linear susceptibility, has a greater ability to polarize in response to the field, thereby reduces the total electric field inside the material (and stores energy).

In nonlinear optics, such as SHG imaging the response of the material is often described as a polynomial expansion of the material polarization **P** in powers of the electric field **E**. The second term defines the SHG.

**P** = ε_0_·(χ^(1)^**E** + χ^(2)^**E**·**E** + χ^(3)^**E**·**E**·**E** +······) (2)

**Figure 2:**
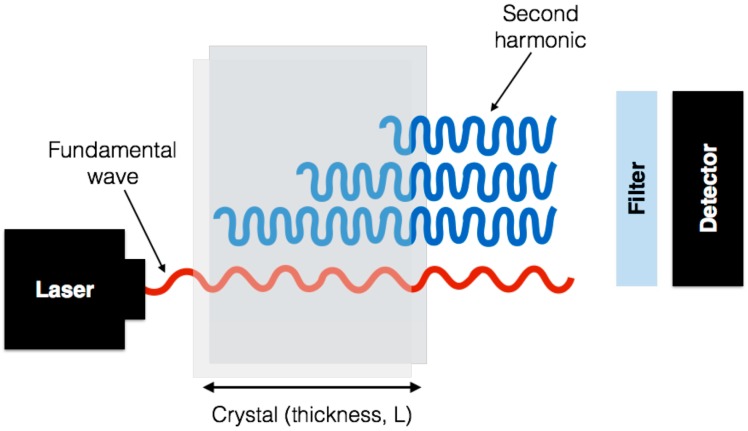
A sketch of the concept of phase matching. The fundamental wave at frequency ω has a well defined phase and amplitude everywhere in the crystal. The induced dipoles all radiate at a frequency 2ω with a phase dictated by the fundamental wave. The picture shows the case where all dipoles radiate in phase in the forward direction so that all contributions add up constructively.

The SHG signal originates from the nonlinear polarization. The incident wave generates dipoles inside the material. These dipoles radiate at twice the frequency of the incident wave. The relative phase of the induced dipoles is fixed because the incident beam has a well-defined amplitude and phase within the material at any given point and time. The SHG signal can be obtained only if the induced dipoles radiate in phase, as illustrated in Figure 2. This phase-matching ensures that the contributions add up constructively from all positions in the material.

There are several factors affecting the magnitude of SHG. It depends quadratically on the intensity of the excitation light, and is affected by the polarization and wavelength of the excitation light. SHG signal can also be dependent on the inherent properties of the material: the nonlinear susceptibility, the phase mismatch between the SHG and the excitation light, and the distribution and orientation of the SHG sources within the focal volume [67,68,69,70].

### 4.2. How Does Fibrillar Collagen Generate SHG Signal

Collagen fibres have a very suitable structure for generating SHG signal. Fibrillar collagen is highly anisotropic and the SHG signal generated is coherently amplified because of the tight alignment of repeating structures within the collagen triple helix and within fibrils. As SHG imaging is dependent on the signal remaining phase-matched within the material, a second harmonic wave generally co-propagates with the excitation beam, resulting in SHG signal in the same, forward direction as the excitation beam [19,69,71]. Typically, between 80% and 90% of the SHG signal from collagen in a tissue sample will propagate in the forward direction, depending on how much the sample scatters light.

**Figure 3:**
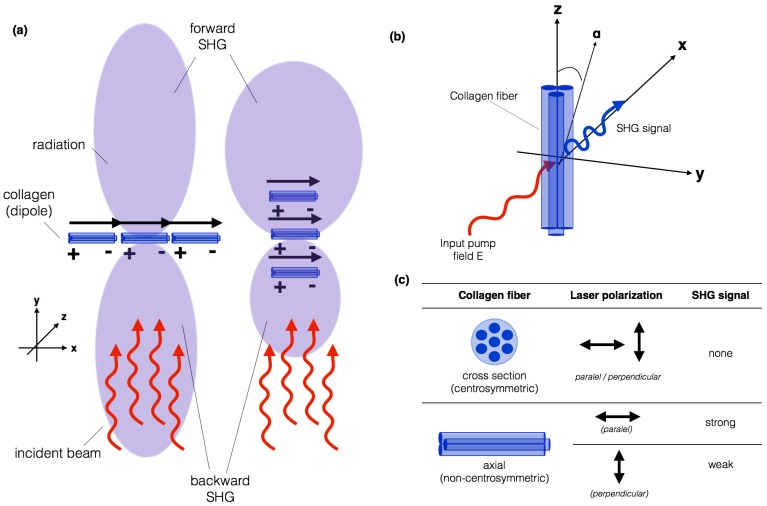
Illustration of (**a**) collagen fibres acting as dipoles, which radiates in all directions except normal to the incident beam; and (**b**) the geometric arrangement of a single collagen fibre relative to an applied electric field. The emitted SHG signal after spectral filtering is shown in blue; (**c**) if the light is polarized along the collagen fibre axis (z), the maximum SHG signal will be observed. On the other hand, if it is polarized perpendicular to the fibre axis (x), the weakest SHG signal will be observed.

A single, 40- to 300-nm collagen fibre would be expected to behave as a single dipole, which radiates in all directions except normal to the incident beam. The peptide bonds within the collagen chains generate a permanent dipole moment, which is a measure of the separation of positive and negative electrical charges within a system, allowing SHG to occur within collagen-rich samples, as shown in Figure 3a.

Figure 3a illustrates how a planar collagen array, normal to the incident beam results in waves in the forward and backward direction being in phase, and therefore radiating strongly in both directions. Little lateral propagation occurs because the wave from one dipole and its neighbor will not be in phase laterally. An array orientated in the direction of the beam will propagate forward, because all dipoles will have the same phase relationship to the excitation beam in the forward direction, regardless of the spacing, but will randomly not be in phase in the backward direction [19,71].

An interesting characteristic of SHG microscopy is that the excited volume within the specimen is elliptical, with the long axis in the direction of the beam. In a collagenous tissue, the overall signal is predominantly propagated in the forward direction, compared to an isolated individual fibre that may radiate SHG signal both forward and backward. Any group of collagen fibres will have more excited dipoles in line with the beam than across it.

An important aspect to consider is that SHG is polarization sensitive. For many sources of SHG, the amount of signal produced is dependent on the polarization state of the incident laser light, relative to the scattering due to the molecular structure. To illustrate this, we shall focus on the polarization sensitivity of SHG from collagen fibrils, as it is the component most imaged by SHG microscopy in biomedical applications [11,13,72,73].

Most studies on the polarization sensitivity of collagen have been carried out on tendon which is composed of highly ordered parallel collagen-type I fibres. The intensity of the SHG signal produced from a collagen sample is dependent on the orientation of the polarization state of the laser excitation light with respect to the fibre axis [72,73,74], as illustrated in Figure 3b. For a linearly polarized laser beam incident on a collagen fibril, the amount of SHG signal produced for different polarization orientations is shown in Figure 3c.

For fibrils lying in the plane perpendicular to the direction of excitation laser light propagation, the amount of SHG produced depends on the angle (α) between the fibre axis (z) and the laser polarization. If the light is polarized along the fibre axis, the maximum SHG signal will be observed [74,75]. On the other hand, if it is polarized perpendicular to the fibre axis, the weakest SHG signal will be observed. This means that the polarization dependence of the SHG signal can be measured to study the orientation of the collagen fibrils within tissue. In the case of the fibre cross-section, due to the fibre structure’s centrosymmetry, no SHG will ever be detected independent of the orientation of the laser polarization. The intensity of the SHG signal also depends on the angle between the collagen fibre and the imaging plane. The intensity of the SHG is maximized when the collagen fibre is in the imaging plane and very low when the fibre is perpendicular to the imaging plane.

The use of polarization analysis in SHG imaging permits the extraction of the tissue’s structural information, including collagen fibre packing. However, for non-polarization resolved SHG imaging, circularly polarized light is preferred, as it will excite all fibre orientations equally. More recent systems use half or quarter wave plates to generate a circular polarized beam which can generate an uniform excitation in all fibre directions (both parallel or perpendicular collagen fibres), and therefore detect the signal generated from all fibres within the tissue, independent of their orientation.

In SHG microscopy the focal area in which the SHG signal is generated has sub-micron dimensions, which is much smaller than the coherence length in collagen. Therefore, the SHG signal should not be significantly reduced by destructive interference. SHG microscopy has proven to be an ideal tool for the analysis and quantification of the spatial arrangement of collagen fibres in tissue [11,13,72,73,74]. This information can be crucial when dealing with complex medical problems, such as atherosclerotic plaque development, fibrosis, airway wall remodeling, etc.

### 4.3. SHG Imaging Systems

**Figure 4:**
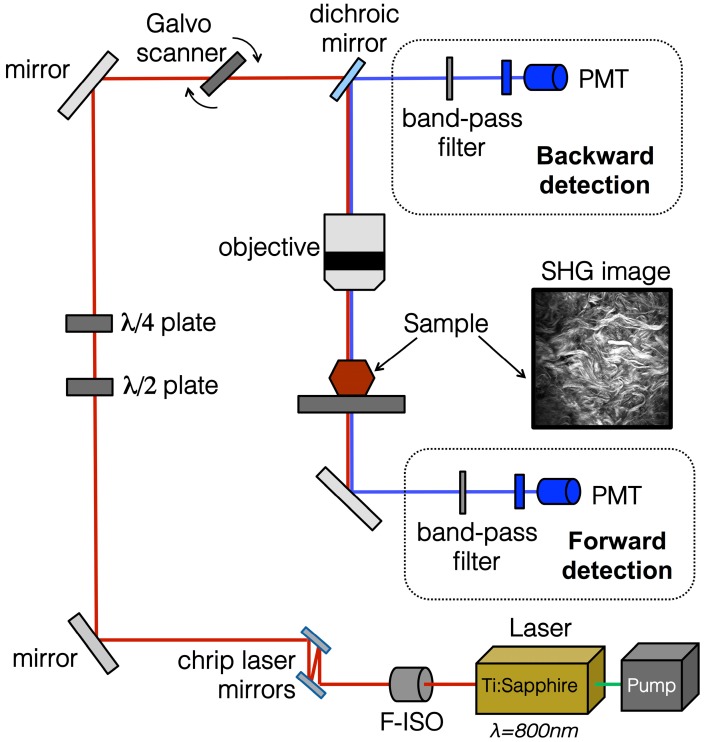
SHG imaging schematic and example image of mouse skin. The optical pathway schematic illustrates the general set-up for SHG imaging. The embedded mouse skin SHG image was taken using a Zeiss 710 confocal microscope was equipped with a Ti:Sa Chameleon multiphoton tunable laser (Coherent, Santa Clara, CA, USA) at 800 nm, a dichroic mirror, a custom filter set (BP:414/46, DC:495, BP:525/50), and a 20× water immersion objective. The resulting image was processed using Zen software (Zeiss Microscopy, Jena, Germany). PMT, photomultiplier tube; F-ISO, Faraday isolator.

There are a number of commercial multi-photon confocal microscopes now available on the market that can be used for SHG imaging (specifically collecting backscatter signal). In general, whether using in-house developed or ready-made equipment, a similar set up is used, as reviewed extensively previously [71] and shown in Figure 4. The basic requirements for a system to image SHG is a scanning confocal microscope equipped with a multi-photon femto-second pulse laser as an excitation source such as the Coherent Chameleon family of titanium:sapphire multi-photon tunable lasers (Coherent Inc., Santa Clara, CA, USA). Figure 4 illustrates the general schematic for the set up for collection of the SHG signal. When imaging the SHG of fibrillar collagen an excitation of 800 nm is used, the light path passes through a linear or circular polarizer, and an objective and reaches the specimen. The backscatter signal is not collected as in normal confocal imaging (such as with a pinhole). Instead, a non-descanned mode is used to collect the entire signal; the photons pass through a long wavelength dichroic mirror and the SHG filter (~410 nm). High gain photomultiplier tubes are then used to collect the SHG signals both in the forward and backward directions. As described above, the addition of polarization to a basic SHG imaging system (Figure 2) provides further information regarding the macrostructural information about the collagen fibre bundles. Therefore, most systems currently available additionally include a λ/2 or λ/4 plate to allow for uniform polarization of the tissue and maximum signal recovery. The maximum depth that can be imaged using such a SHG imaging system depends on the characteristics of the tissue. For example, the melanin in pigmented skin will burn at the laser power required to image more than ~50 μm into the superficial side, but if excised and flipped over the skin can be imaged ~300 μm into the dermal surface.

### 4.4. Recent In Vivo Instrumentation Advances

Multimodal nonlinear optical (NLO) laser-scanning microscopes have been key to visualizing several specific biomolecules without the need of any specific tissue preparation and providing superior spatial and biochemical specificity. Coherent anti-Stokes Raman scattering (CARS), stimulated Raman scattering (SRS), two-photon-excitation fluorescence (TPEF), SHG and third harmonic generation (THR) have been extensively used in biomedical research. However, one of the biggest challenges faced by scientists are related to miniaturizing these laser-based microscopes to a point where these techniques could be easily used in clinic and in vivo.

Several groups have been intensively working towards this goal [76,77,78]. Crisafi et al. have presented the design of a multimodal NLO laser-scanning microscope on a compact fibre-format, integrating three NLO modalities (CARS, SRS, and TPEF) [79]. Their proposed system offers for the first time the possibility to develop a NLO microscope using off-the-shelf components, providing a cost-effective alternative to commercial systems. Song et al. were successful in adding SHG into the mix, developing a fully integrated multimodal microscopy that can provide photoacoustic (optical absorption), TPEF and SHG information from tissue in vivo [80]. The authors were able to visualize the cortex of a mouse and SHG was successfully used to reveal complementary tissue microstructures. Other groups, including Atsuta et al., were able to monitor collagen changes in human skin, by using fibre optic delivery of the pulse light, making a compact SHG microscope enclosed into a lens tube system [81]. This system provided the flexibility needed to perform measurements of several regions in the human skin.

Finally, another recent application of miniaturized SHG was proposed by Schnitzer et al. [82,83]. They have combined previous ideas of SHG imaging sarcomere organizations in human patients in vivo. They developed a miniaturized microscope which can be attached to the desired region (or limb), and therefore acquire information on sarcomere length coupled with electrical stimulation from normal patients, from stroke patients on both affected and unaffected limbs, and from patients recovering from injury. This application of SHG microscopy represents an important step towards the first true in vivo image system able to monitor muscle structure in patients.

## 5. Alterations in Fibrillar Collagens in the Disease State

**Figure 5:**
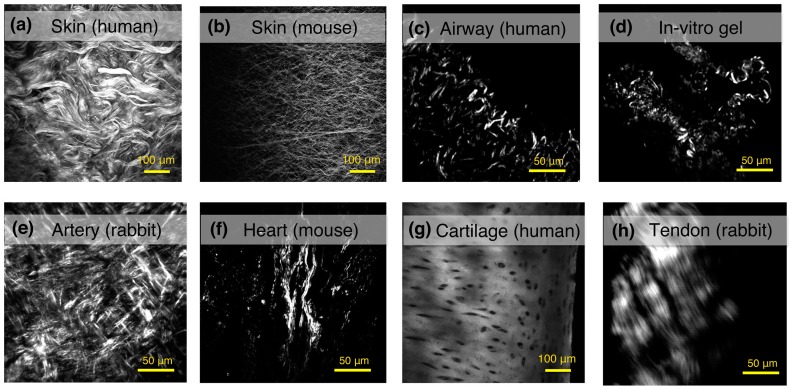
Examples of label-free SHG images from different tissue and specimens. The collagen network can be observed at the dermal layer of (**a**) human skin; or (**b**) mouse skin. The collagen deposition in human airways from a (**c**) human healthy donor; and (**d**) in vitro collagen gel model, showing fibrillar collagen synthesized by human fibroblasts from airways; (**e**) adventitia layer of a healthy aorta artery (rabbit); and (**f**) scar tissue formation in infarcted hearts from mouse; (**g**) human cartilage; and (**h**) tendons from rabbits can also be assessed using SHG imaging.

SHG imaging has been used to study a number of tissues and assess the quality of the fibrillar collagen in both the normal and disease state. In Figure 5, we present some examples of label-free SHG images from different specimens of normal tissues. This imaging technology is capable of not only providing a snap-shot of the collagen structure, which is useful in itself, but also of helping to ascertain key biologically relevant information about disease pathology that will be key to furthering our understanding of the pathological process in many fibrotic diseases. Table 1 summarizes the breadth of studies that are possible in skin, lung, cardiovascular, tendon and ligaments, and eye tissues using SHG imaging in biomedical research (discussed in more detail below).

### 5.1. Skin

The skin is composed of three main layers: the epidermis, dermis and hypodermis. The epidermis is a thin layer of epidermal cells covered by the stratum corneum and provides the barrier function of the skin. The deeper dermal layer is primarily collagenous and provides structural support for cells and the dermal appendages (sabaceous glands, hair follicles, etc.), as well as the mechanical properties (strength, flexibility, distensibility, etc.) of the skin [101]. A large proportion of the cells within the dermis are mesenchymal in origin including interstitial fibroblasts, which maintain the ECM and the hair follicle dermal stem cells, which expand during injury to contribute to the interstitial fibroblast population [102]. The hypodermis is essentially highly vascularized adipose tissue. SHG imaging has proven useful in determining the directionality and organization of the fibrillar collagen in the skin, in both health and disease. Compared to cross-sections imaged after histochemical staining with Picro-Sirius Red stain or immunostaining for collagen, the 3D images that are created using SHG imaging of the whole tissue allow for determination of the arrangement of the collagen content in the dermis.

Recent studies have used SHG to distinguish between normal and pathologically scarred skin, suggesting potential diagnostic use for SHG imaging in the clinic [86,103]. Studies using SHG imaging have illustrated that the overall isotropic nature of dermal collagen provides the necessary distensability for mobility (to avoid puncture and for joint motion). Of particular note is that the proportion of collagen fibres that are aligned increases in keloid and surgical scar tissue [104,105,106]. The collagen fibre bundles in scarred skin after grafting have been shown to be straighter and thinner [85]. These factors appear to contribute to the decrease in the tensile strength of the scar compared with normal skin [85,107].

### 5.2. The Lung

The lung is formed from a dichotomous branching structure of airways that enables airflow that enters the nose and mouth to be directed to the 600 million terminal alveoli structures where gas exchange occurs. In terms of fibrillar collagens, the developing lung contains primarily fibrillar collagens I and III, which are deposited primarily by lung fibroblasts during the canalicular stage, in which the respiratory airways and alveolar ducts are formed, preceding the bulk of elastogenesis [108,109]. From the late stages of gestation to adult life, the collagen content in the lung increases five-fold. Specifically, in the rabbit lung, it has been shown during maturation that rapid growth after birth leads to a 15% increase in collagen synthesis relative to the rate of total protein synthesis which declines after two months [110]. In bronchopulmonary dysplasia (BPD) patients and animal models of BPD, ECM cross-linking enzymes are deregulated and aberrant late lung development blocks alveolarization, suggesting that perturbed ECM cross-linking may impact alveolarization [111]. Further, in many obstructive lung diseases that involve airway fibrosis such as asthma, chronic obstructive pulmonary disease (COPD) and idiopathic pulmonary fibrosis (IPF), alterations in fibrillar collagens due to deposition or turnover are the primary focus of research.

To date, SHG imaging of fibrillar collagen has primarily been used to confirm fibrotic regions in human IPF tissues [112] and mouse IPF models [113] identified by histological stains (Masson’s trichrome). The technique has also been used to identify differences in the collagen organization in emphysematous COPD lung tissues compared to control donors [114,115]. In terms of quantification of SHG, signal intensity has previously been used to quantify the fibrosis-related increase in fibrillar collagen in a mouse model of pulmonary fibrosis [112], a human-mouse xenograft model of airway epithelial-induced fibrosis in asthma [116] and an in vitro collagen-gel contraction model using lung fibroblasts from COPD patients [117]. The SHG signal intensity has also been used to compare the ratio of collagen and elastin (measured by TPEF) in COPD lungs [115]. Using a standardized approach Tijn et al. reported on the potential to use forward SHG (F-SHG)/backward SHG (B-SHG) ratios as an approach to determine the amount of disorganized collagen in the airways of patients with COPD compared to controls [96]. Most recent studies have used the F-SHG/B-SHG ratio propagation SHG signal to demonstrate that the collagen microstructure is altered in regions of normal tissue and usual interstitial pneumonia, with increased mature fibrillar collagen compared to a reduced mature elastin fibre content. However, only preliminary data presented in abstract form has applied texture analysis to the human airways to demonstrate that collagen fibres are more disorganized in asthmatic airways compared to controls throughout the entire airway tree [118].

### 5.3. Cardiovascular System

Arteries are mainly composed of soft collagenous tissue; they serve as an elastic reservoir to transform the high pressure, pulsatile output into a flow with moderate fluctuations [119]. The vessel walls of large arteries have a number of structural features in common, although structural variations between the various arteries do exist. Fibrillar collagen is the ubiquitous load-bearing element which confers the macroscopic mechanical responses of the arterial wall, which are highly nonlinear, elastic and anisotropic [119]. The arrangement of collagen fibres in concentric anisotropic layers leads to the anisotropic mechanical behavior of arterial tissues. As the ECM is primarily composed of fibrillar collagen, in the arteries, it provides the majority of cellular attachment. Collagen tearing and defects result in disease and changes in the biomechanical behavior of the arterial tissue [14,42]. These alterations can result from the buildup of arterial plaques (or atherosclerosis), consisting of extracellular deposits of low-density lipids (LDLs), that gradually develop over a period of many years [120,121]. Current available clinical methods (ultrasound and magnetic resonance imaging) lack sufficient resolution to follow the structural changes in the ECM composition of the arterial lumen, as this requires imaging on the micron-scale [122,123]. SHG imaging may be suitable for identifying changes in arterial wall structure and composition as it could identify areas at risk of rupture earlier than currently available techniques, leading to the development of novel treatments specific for these alterations [14,124,125]. There are current limitations to SHG imaging in the cardiac system, as it has been shown ineffective at imaging arterial branch points, which are thought to be most susceptible to plaque formation due to local wall stress and heterogeneities of blood flow [90,91,93]. Studies using multimodal SHG microscopy have shown that mechanical considerations do not sufficiently convey the risk of rupture, but that local changes in biochemical composition must also be considered, which would not have been discerned by other clinical imaging modalities and standard histology alone [90,91,92].

In addition to arteries, SHG imaging of collagen has been used for the characterization of cardiac scar tissue formed due to myocardial infarction [40]. Mostaço-Guidolin et al. [40] showed significant reduction of collagen deposition in the adipose-derived stem cells (ASCs)-treated infracted heart. In the ASCs-treated infarcted myocardium, SHG imaging revealed highly-directional and organized collagen fibres compared to the un-treated infarcted myocardium, in which the collagen was significantly less organized.

### 5.4. Tendons and Ligaments

Tendons and ligaments facilitate a wide range of joint motion and considerable weight and energy savings associated with locomotor movement [87,126]. Tendons transmit forces between the muscle and bone, providing function, while ligaments provide bone to bone transmission of force, providing stability [87,126,127]. Although these two structures have different functions, both of them have similar characteristics. Specifically, both tendons and ligaments are predominantly composed of fibrillar collagen type I molecules arranged as fibrils, fibres, fibre bundles and fascicles [126,127]. The cellular components of tendons and ligaments consist of mature fibroblast and fibrocytes, although these are in low abundance and the majority of the tissue in both is composed of collagens and some glycosaminoglycans and elastic fibres [87,127,128,129]. A number of studies have now focused on the structural and molecular organization of the ECM in tendons and ligaments [87,126,127,128]. Tendons and ligament defects have largely been explained by abnormal collagen fibrillogenesis [130,131]. SHG imaging is capable of evaluating collagenase-induced tendon injury [89,94,132], with the ability to clearly differentiate normal and injured tendon collagen fibre organization. Injured tendons display less ordered collagen fibres in comparison to normal tendons. Biomechanically, abnormalities in crimp structure (which allow longitudinal elongation of tendons in response to loads) and collagen fibre organization result in a loss of performance of normal functions and thus degrade a subject’s ability to move. SHG microscopy has been shown to be more sensitive to assessing tendon injury than standard clinical measurements using histology and light microscopy.

### 5.5. The Eye

The stroma of the eye is composed mainly of collagen type I, however common methods that are used for imaging the corneal epithelium (confocal microscopy) are ineffective for imaging this collagenous tissue [14,133]. Furthermore, due to the potential for permanent injury from resection of in vivo tissues, traditional histological techniques are not an option for diagnosing pathologies of the stroma. Because the collagenous composition of corneal stroma, SHG microscopy is an ideal option for the label-free imaging and diagnosis of pathological conditions [24]. For example, Tan et al. characterized collagen disruption in infectious keratitis using SHG imaging and simultaneously used two-photon fluorescence to identify infectious pathogens (bacterial, fungal and protozoan) ex vivo [97]. SHG imaging cannot compete with electron microscopy in terms of resolution, however under physiological conditions in vivo the structure of cornea can be investigated using SHG imaging [14,24,97].

### 5.6. In Vitro Models: Collagen Gels

Cellularized collagen gels are a common model used to understand several biological processes involving the interactions of cells with collagen [20,34,100,117,134,135]. Collagen gels have been successfully imaged by SHG microcopy, as they can provide information on structural rearrangements in the 3-D structure of the collagen matrix, which is modified by various cellular physiological processes [100]. Ajeti et al. [20] proposed the use of collagen gels, consisting of mixtures of collagen type I and type V isoforms to serve as a model of the ECM during cancer invasion in vivo. Using several metrics from SHG images, they found that SHG imaging was sensitive to the incorporation of Collagen V into Collagen I fibrils. They developed SHG microscopy as a tool to discriminate Collagen I/Collagen V composition in tissues to characterize and follow breast cancer invasion [20]. Another example of collagen gel characterization using intensity based analysis of SHG images was presented by Campbell et al., who concluded that defective collagen I remodeling and contraction was a feature of COPD parenchymal fibroblasts compared to fibroblasts derived from normal donors [117]. Finally, in tissue engineering, collagen gels and SHG images have played a vital role providing insights about the relationship between the microstructure and tissue bulk mechanical properties [134]. SHG has been proven to be an ideal non-invasive tool for examining collagen microstructure, cellularity and crosslink content in gels in both in vitro models and biological tissue.

## 6. Quantitative Image Analysis Methods

SHG microscopy has been a very powerful tool in biomedical research. However, most published SHG imaging work has described collagen organization without focusing on the quantitative measures which are possible to implement to characterize SHG images. In several studies, pathological conditions were described using empirical observations derived from collagen SHG images. While the ability to track associations between collagen SHG images and pathology is important, it is equally important to have the ability to track such correlations using quantifiable measures for objective comparison. Quantitative SHG imaging analysis methods have largely relied on image pixel-counting techniques, similar to those applied to histological tissue images. Several SHG collagen imaging studies have recently proposed novel methodologies for quantifying the features of SHG images.

Although commercially available laser scanning microscopes are supplied with software with some rudimentary data analysis capabilities, many groups have been working on developing methods to quantify the fibrillar structures detected in SHG images. Many external software packages have been successfully used for analysis of SHG data, including the measurement of intensities, the application of a threshold for measuring fibre lengths and division for F-SHG/B-SHG analysis. ImageJ or Fiji can run these analyses, or the can be automated using MATLAB or LabVIEW. Commercial packages including Improvision and Imaris are capable of rendering 3D images, as are some plugins especially written for Fiji. We present below the most commonly used analysis methods for SHG data; however, we would like to note that, while these processes are integral to measuring fibre length and distribution, they are not specific to SHG image analysis.

### 6.1. Intensity-Based Analysis

Intensity-based image analysis is the most straightforward form of quantification. The signal collected in each individual detector is associated with a specific pixel. Each pixel receives a value which can be associated to the signal intensity, and therefore, the amount of collagen deposited in that specific region. It is important to highlight that intensity-based analysis takes into account the intensities of individual pixels, and they are considered independently from their neighboring pixels [40]. Other than the absolute intensity value detected by each region of the sensor (which will later become the image’s pixels), some first-order statistics can describe the gray levels (or intensities) of the histogram corresponding to an image. Among these parameters, the most commonly applied in SHG data analysis are the mean, standard deviation, integrated density, skewness, and kurtosis. The mean and integrated densities provide measures of the overall lightness/darkness of the image, while the standard deviation describes its overall contrast. These measures can be associated to the amount of collagen fibres, which is proportional to the detected SHG signal. The skewness quantitatively evaluates the asymmetry of the shape of the distribution of pixel intensities around the mean value of the histogram, while kurtosis measures the peakedness of the distribution relative to the length and size of the histogram tails [40]. Skewness and kurtosis can be useful when aiming to detect fibre features such as edges and how distinct they are from a certain background. In digital image processing, kurtosis values can be interpreted in combination with noise and resolution measurements. On the other hand, skewness tend to be positive in darker and glossier surfaces than lighter and matte surfaces, being therefore useful in making judgments regarding the fibre surfaces.

### 6.2. Forward–Backward SHG-Signal

To exploit the coherence of SHG and thus extract sub-resolution feature information, the SHG microscope could be set up with both F-SHG and B-SHG collection channels. The microscope’s transmission pathway enables us to collect the F-SHG signal. This mode allows one to collect the majority of the SHG signal. To acquire B-SHG, the use of the confocal laser scanning head is necessary. It is important to highlight that to distinguish between TPEF and SHG signal it is necessary to perform a synchronous spectrum. In terms of quantitative analysis, the structural differences observed between the F-SHG and B-SHG images have not been well studied [136]. F-SHG/B-SHG ratio measurements are one of the most common quantitative measures presented in SHG image analysis. Some groups have claimed to be able to differentiate collagen types by calculating F-SHG/B-SHG ratio measurements. However, more rigorous experiments with standardized samples must be performed and correlated with traditional techniques, such as immunohistochemistry, before we can attribute this capability to SHG microscopy. F-SHG/B-SHG ratio measurements can be useful for assessing the fibre orientation content. Laterally oriented fibres appear primarily in the backward direction, whereas axial oriented fibres appear primarily in the forward direction. To successfully extract information from this measure, the relative collection efficiencies of the two detection pathways (transmission and confocal), including the detectors, need to be calibrated for each objective/condenser combination. This procedure must be done with isotropic emitters, such as KDP (monopotassium dihydrogen phosphate) crystal. One alternative to F-SHG/B-SHG images is the use of circularly polarized light; it can be obtained by placing a quarter-wave plate just before the objective. Circularly polarized nonlinear optics has the ability to quantify molecular symmetry after the acquisition of a minimal number of images using exclusively intensity information [137]. It can therefore be exploited to quantify the molecular alignment in arterial collagen with a single image during mechanical loading. The SHG intensity with circularly polarized light is not sensitive to the absolute orientation of the scattered signals, thus enabling characterization of their relative degree of order within a single image per tissue state.

### 6.3. Polarization

Polarization-resolved SHG is an alternative that can be used to extract information beyond simple visualization of fibre lengths or by pixel intensity. Polarization-resolved SHG is a form of measurement, which analyses the signal intensity as a function of laser polarization. Examples of this type of SHG measurement have been presented by some authors [75,138], where, by analyzing the signal anisotropy for constant linear polarization excitation, they were able to gather data on the protein helical pitch angle and the dipole alignment angle, respectively.

As the SHG signal generated by collagen fibres is highly dependent on their orientation, when working with polarization-resolved SHG, one can easily create a gamut of images showing the preferred fibre orientation within a certain region. As a general guideline, in the first measurement, the laser polarization is aligned with the long axis of a collagen fibre(s), and then rotated through 180${}^{\circ }$, where the intensity of these successive images is recorded. This collection of images can be used to verify changes occurring during collagen remodeling in scar formation, as the collagen fibres in this case tend not to present a preferable orientation.

### 6.4. Transform-Based Methods

Another way to quantitatively extract information from SHG images is by applying transform-based image analysis techniques. These techniques represent an image in a space whose coordinate system has an interpretation that is closely related to the characteristics of a texture, using the spatial frequency properties of the pixel intensity variations. Several methods are available and the success of these methods will strongly depend on the type of transform used to extract textural characteristics from the image and the final goal of the overall analysis. Methods based on Fast Fourier Transform (FFT) usually perform poorly in practice, due to its lack of spatial localization. However, Indhal and Næs [139] illustrated the use of spectra from 2-D FFT magnitude images for textural feature extraction, which can be used to determine the percentage of pixels and therefore collagen fibre bundles with the same alignment [85]. Gabor filters provide means for better spatial localization. Features derived from a set of Gabor filters have been widely used in texture analysis for image segmentation [140,141]. However, their usefulness is limited in practice because there is usually no single filter resolution at which one can localize a spatial structure in biological samples. Wavelet transform methods of feature extraction offer several advantages over FFT and Gabor-based methods. Wavelets have been used to characterize texture and to treat the problems of texture segmentation and classification [142,143]. FFT has been by far the most used method to characterize SHG images due to its simplicity and availability in several image analysis software packages. FFT has been able to show differences between different types of tissue and/or conditions, such as cancer-stage differentiation, scar formation and collagen remodeling in skin diseases. FFT analysis can be useful when combined with F-SHG/B-SHG or polarization-resolved SHG images, as it provides a quantitative measure of fibre orientation. However, further exploration of the capability of wavelets to aid in the interpretation of SHG images is still necessary.

### 6.5. Texture Analysis

Texture analysis has always been a powerful tool in the analysis of bio-medical imaging, remote sensing and industrial inspection, and its outputs are mainly classification, segmentation, and synthesis. Textures are very diverse and the approaches for analyzing them are likewise very diverse, and mainly differ from each other by the method used for extracting textural features. Texture analysis techniques, also called higher order statistics, primarily describe characteristics of regions in an image through higher-order measures of their grayscale histograms. The most robust and frequently cited method for texture analysis is based on extracting various textural features from a gray level co-occurrence matrix (GLCM) [144]. The GLCM approach is based on the use of second-order statistics of the grayscale image histograms. Entropy, inverse difference moment (IDM), energy, inertia, entropy and correlation were reported as the most suitable textural parameter to evaluate collagen changes [40,117,118,144]. Entropy has been associated with the degree of fibre organization, and therefore can be very useful for the characterization of several tissue and conditions [144]. Alternatively, the run length matrix (RLM) texture analysis approach characterizes coarse textures as having many pixels in a constant gray level run and fine textures as having few [145]. For the purpose of collagen characterization, the choice between GLCM and RLM is clear: GLCM provides more flexibility and is able to provide quantitative measures of a varied of fibre features.

## 7. Limitations

There is no question that SHG microscopy is a powerful tool for examining biological tissue. However, as with any technology, it also presents limitations. As is the case with other NLOM modalities, SHG needs a laser as a light source. Most of the commercial femto-seconds lasers (needed to generate SHG signal) are based on Ti:Sapphire laser sources. The price of Ti:Sapphire lasers have decreased over the years, but are still quite an investment. One recent advance in this regard is the use of solid-state pump lasers, as they are less expensive, more compact and reliable sources for NLOM.

Another major factor that significantly affects making SHG microscopy applicable for broader use is the availability of compact systems small enough to fit into a catheter-sized probe that can be used to acquire in vivo images, as well as fully automated and maintenance-free systems. This next step for laser and NLOM development will allow these techniques to be used in routine, biological and clinical environments without the need for laser physicists.

The limited penetration depths (100–300 μm with laser excitation in the 800–1000 nm range) make SHG microscopy not suitable for certain applications, such as when the region of interested is located centimeters within the tissue or organ. Using conventional optical geometry, the spatial resolution of SHG microscopy is also limited by the diffraction limit, which is related to the wavelength (several hundred nm) of the incident waves. Super-resolution NLOM is starting to become available and this limitation should soon be overcome by the next generation of microscopes.

Lastly, for biological applications, the largest limitation to SHG microscopy is its ability to assess only a small number of structural proteins or harmonophores. In addition, to fibrillar collagens, this includes actin-myosin complexes, microtubules, centrosomes and mitotic spindles in cells (not discussed here). To date there are no methods available that can distinguish between fibrillar collagen types, which could greatly advance the field of wound repair and regeneration biology.

## 8. Conclusions

Understanding scar formation (fibrillar collagen formation) over time in humans has huge potential to deliver new therapeutics for correct wounds. Without the need for labeling, SHG imaging is particularly suitable for in vivo studies of collagenous connective tissue, partially due to strong contrast and large sensing depth. In future, the development of SHG endoscopes comprised of a pulsed laser source, a fibre-optic catheter, and a microelectromechanical systems (MEMS)-based scanning head, could offer large potential to clinical research. This would enable achievable penetration depths of 100–200 micrometers to analyze scar tissue non-invasively in human subjects using current clinical endoscopy techniques. Such a device could aid in assessment of scar tissue and enable new protocols to non-invasively monitor the efficiency of therapeutics and their effects on tissue function in vivo.

## Figures and Tables

**Table 1:** Representative examples of Second Harmonic Generation (SHG) applications.

Organ/Tissue	Key Conclusions	Representative Reference
Skin	Uniquely shows changes in collagen assembly upon thermal damage. Highly valuable for diagnosing and screening early melanocytic lesions. SHG to auto-fluorescence aging index of dermis (SAAID) can be a good indicator of the severity of photoaging; Distinct morphological differences in melanoma compared with melanocytic nevi. Used to distinguish between scar (keloid and hypertrophic) and normal tissue Shows alteration in collagen structure in scar tissue after grafting	[23,30,84,85,86]
Tendon and Ligaments	Good at determining the orientation of collagen fibrils in the fascicle and the ratio γ between the two independent elements of the second-order nonlinear susceptibility tensor; Normal cartilage reveals a consistent pattern of variation in fibril orientation with depth. In lesions, the pattern is severely disrupted and there are changes in the pericellular matrix; The differences in collagen fibre organization between normal and injured tendon indicate that the organization of collagen fibres is regularly oriented in normal tendons and randomly organized in injured tendons.	[75,87,88,89]
Cardiovascular	SHG shows that collagen plaques intermingle with elastin; Based on a measure of the collagen/elastin ratio, plaques were detected with a sensitivity of 65% and specificity of 81%. Furthermore, the technique gives detailed information on the structure of the collagen network in the fibrous cap; Single parameter based on intensity changes derived from multi-channel nonlinear optical (NLO) images can classify plaque burden within the vessel; Using the optical index for plaque burden (OIPB) it was possible to differentiate between healthy regions of the vessel and regions with plaque, as well as distinguish plaques relative to the age; Texture analysis based on first-order statistics (FOS) and second-order statistics such as gray level co-occurrence matrix (GLCM) extracted SHG image features that are associated with the structural and biochemical changes of tissue collagen networks.	[28,40,90,91,92,93]
Lung	Significant difference in collagen organization in airway tissue between chronic obstructive pulmonary disease (COPD) and non-diseased; High resolution images can be generated without the need of staining and tissue damage; Fibrillar collagen’s subresolution structure is altered in usual interstitial pneumonia versus cryptogenic organizing pneumonia and healthy lung	[94,95,96]
Eye	SHG can delineate the stroma from other corneal components. SHG imaging revealed that corneal collagen fibrils are regularly packed as a polycrystalline lattice, accounting for the transparency of cornea. In contrast, scleral fibrils possess inhomogeneous, tubelike structures with thin hard shells, maintaining the high stiffness and elasticity of the sclera.	[24,97]
In-vitro models	Self-assembled fibrillar gels can be imaged by SHG SHG can characterize differential microscopic features of the collagen hydrogel that are strongly correlated with bulk mechanical properties Depending on the collagen source, in vitro models yield homogeneous fibrillar texture with a quite narrow range of pore size variation, whereas all in vivo scaffolds comprise a range from low- to high-density fibrillar networks and heterogeneous pore sizes within the same tissue.	[34,98,99,100]

## Abbreviations

SHG	Second harmonic generation
THG	Third harmonic generation
B-SHG	Backward second harmonic generation
F-SHG	Forward second harmonic generation
Col	Collagen
COPD	Chronic obstructive pulmonary disease
CARS	Coherent anti-Stokes raman spectroscopy
MEMS	Microelectromechanical system
GLCM	Gray level co-occurrence matrix
IDM	Inverse difference moment
RLM	Run length matrix
AMT	Angle Measure Technique
FFT	Fast Fourier Transform
ECM	Extracellular matrix
3D	3-dimension
LDL	Low density lipids
ASC	Apdipose stem cell
NLOM	Non-linear optical microscopy
TPEF	Two photon electron fluorescence
BPD	Bronchopulmonary dysplasia

## References

[1] Konigova R., Rychterova V. (2000). Marjolin’s ulcer. Acta Chir. Plast..

[2] Trent J.T., Kirsner R.S. (2003). Wounds and malignancy. Adv. Skin Wound Care.

[3] Aarabi S., Longaker M.T., Gurtner G.C. (2007). Hypertrophic scar formation following burns and trauma: New approaches to treatment. PLoS Med..

[4] Singer A.J., Clark R.A. (1999). Cutaneous wound healing. N. Engl. J. Med..

[5] Colwell A.S., Longaker M.T., Lorenz H.P. (2003). Fetal wound healing. Front. Biosci..

[6] Brem H., Tomic-Canic M. (2007). Cellular and molecular basis of wound healing in diabetes. J. Clin. Investig..

[7] Sen C.K., Gordillo G.M., Roy S., Kirsner R., Lambert L., Hunt T.K., Gottrup F., Gurtner G.C., Longaker M.T. (2009). Human skin wounds: A major and snowballing threat to public health and the economy. Wound Repair Regen..

[8] Reish R.G., Eriksson E. (2008). Scars: A review of emerging and currently available therapies. Plast. Reconstr. Surg..

[9] Richmond N.A., Lamel S.A., Davidson J.M., Martins-Green M., Sen C.K., Tomic-Canic M., Vivas A.C., Braun L.R., Kirsner R.S. (2013). US-National Institutes of Health-funded research for cutaneous wounds in 2012. Wound Repair Regen..

[10] Mertz J. (2004). Nonlinear microscopy: New techniques and applications. Curr. Opin. Neurobiol..

[11] Williams R.M., Zipfel W.R., Webb W.W. (2001). Multiphoton microscopy in biological research. Curr. Opin. Chem. Biol..

[12] Yue S., Slipchenko M.N., Cheng J.X. (2011). Multimodal Nonlinear Optical Microscopy. Laser Photon. Rev..

[13] Zipfel W.R., Williams R.M., Webb W.W. (2003). Nonlinear magic: Multiphoton microscopy in the biosciences. Nat. Biotechnol..

[14] Campagnola P. (2011). Second harmonic generation imaging microscopy: Applications to diseases diagnostics. Anal. Chem..

[15] Campagnola P.J., Loew L.M. (2003). Second-harmonic imaging microscopy for visualizing biomolecular arrays in cells, tissues and organisms. Nat. Biotechnol..

[16] Campagnola P.J., Millard A.C., Terasaki M., Hoppe P.E., Malone C.J., Mohler W.A. (2002). Three-dimensional high-resolution second-harmonic generation imaging of endogenous structural proteins in biological tissues. Biophys. J..

[17] Zipfel W.R., Williams R.M., Christie R., Nikitin A.Y., Hyman B.T., Webb W.W. (2003). Live tissue intrinsic emission microscopy using multiphoton-excited native fluorescence and second harmonic generation. Proc. Natl. Acad. Sci. USA.

[18] Masihzadeh O., Schlup P., Bartels R.A. (2010). Label-free second harmonic generation holographic microscopy of biological specimens. Opt. Express.

[19] Pfeffer C.P., Olsen B.R., Ganikhanov F., Legare F. (2008). Multimodal nonlinear optical imaging of collagen arrays. J. Struct. Biol..

[20] Ajeti V., Nadiarnykh O., Ponik S.M., Keely P.J., Eliceiri K.W., Campagnola P.J. (2011). Structural changes in mixed Col I/Col V collagen gels probed by SHG microscopy: Implications for probing stromal alterations in human breast cancer. Biomed. Opt. Express.

[21] Cicchi R., Massi D., Sestini S., Carli P., De Giorgi V., Lotti T., Pavone F.S. (2007). Multidimensional non-linear laser imaging of Basal Cell Carcinoma. Opt. Express.

[22] Conklin M.W., Eickhoff J.C., Riching K.M., Pehlke C.A., Eliceiri K.W., Provenzano P.P., Friedl A., Keely P.J. (2011). Aligned collagen is a prognostic signature for survival in human breast carcinoma. Am. J. Pathol..

[23] Dimitrow E., Ziemer M., Koehler M.J., Norgauer J., Konig K., Elsner P., Kaatz M. (2009). Sensitivity and specificity of multiphoton laser tomography for in vivo and ex vivo diagnosis of malignant melanoma. J. Investig. Dermatol..

[24] Han M., Giese G., Bille J. (2005). Second harmonic generation imaging of collagen fibrils in cornea and sclera. Opt. Express.

[25] Kirkpatrick N.D., Brewer M.A., Utzinger U. (2007). Endogenous optical biomarkers of ovarian cancer evaluated with multiphoton microscopy. Cancer Epidemiol. Biomark. Prev..

[26] Kwon G.P., Schroeder J.L., Amar M.J., Remaley A.T., Balaban R.S. (2008). Contribution of macromolecular structure to the retention of low-density lipoprotein at arterial branch points. Circulation.

[27] Lacomb R., Nadiarnykh O., Campagnola P.J. (2008). Quantitative second harmonic generation imaging of the diseased state osteogenesis imperfecta: Experiment and simulation. Biophys. J..

[28] Le T.T., Langohr I.M., Locker M.J., Sturek M., Cheng J.X. (2007). Label-free molecular imaging of atherosclerotic lesions using multimodal nonlinear optical microscopy. J. Biomed. Opt..

[29] Lin S.J., Jee S.H., Kuo C.J., Wu R.J., Lin W.C., Chen J.S., Liao Y.H., Hsu C.J., Tsai T.F., Chen Y.F. (2006). Discrimination of basal cell carcinoma from normal dermal stroma by quantitative multiphoton imaging. Opt. Lett..

[30] Lin S.J., Wu R., Tan H.Y., Lo W., Lin W.C., Young T.H., Hsu C.J., Chen J.S., Jee S.H., Dong C.Y. (2005). Evaluating cutaneous photoaging by use of multiphoton fluorescence and second-harmonic generation microscopy. Opt. Lett..

[31] Lo W., Teng S.W., Tan H.Y., Kim K.H., Chen H.C., Lee H.S., Chen Y.F., So P.T., Dong C.Y. (2006). Intact corneal stroma visualization of GFP mouse revealed by multiphoton imaging. Microsc. Res. Tech..

[32] Nadiarnykh O., LaComb R.B., Brewer M.A., Campagnola P.J. (2010). Alterations of the extracellular matrix in ovarian cancer studied by Second Harmonic Generation imaging microscopy. BMC Cancer.

[33] Provenzano P.P., Eliceiri K.W., Campbell J.M., Inman D.R., White J.G., Keely P.J. (2006). Collagen reorganization at the tumor-stromal interface facilitates local invasion. BMC Med..

[34] Raub C.B., Suresh V., Krasieva T., Lyubovitsky J., Mih J.D., Putnam A.J., Tromberg B.J., George S.C. (2007). Noninvasive assessment of collagen gel microstructure and mechanics using multiphoton microscopy. Biophys. J..

[35] Sahai E., Wyckoff J., Philippar U., Segall J.E., Gertler F., Condeelis J. (2005). Simultaneous imaging of GFP, CFP and collagen in tumors in vivo using multiphoton microscopy. BMC Biotechnol..

[36] Schenke-Layland K., Xie J., Angelis E., Starcher B., Wu K., Riemann I., MacLellan W.R., Hamm-Alvarez S.F. (2008). Increased degradation of extracellular matrix structures of lacrimal glands implicated in the pathogenesis of Sjogren’s syndrome. Matrix Biol..

[37] Strupler M., Pena A.M., Hernest M., Tharaux P.L., Martin J.L., Beaurepaire E., Schanne-Klein M.C. (2007). Second harmonic imaging and scoring of collagen in fibrotic tissues. Opt. Express.

[38] Sun W., Chang S., Tai D.C., Tan N., Xiao G., Tang H., Yu H. (2008). Nonlinear optical microscopy: Use of second harmonic generation and two-photon microscopy for automated quantitative liver fibrosis studies. J. Biomed. Opt..

[39] Ko A.C., Ridsdale A., Mostaço-Guidolin L.B., Major A., Stolow A., Sowa M.G. (2012). Nonlinear optical microscopy in decoding arterial diseases. Biophys. Rev..

[40] Mostaço-Guidolin L.B., Ko A.C., Wang F., Xiang B., Hewko M., Tian G., Major A., Shiomi M., Sowa M.G. (2013). Collagen morphology and texture analysis: From statistics to classification. Sci. Rep..

[41] Wang H.W., Langohr I.M., Sturek M., Cheng J.X. (2009). Imaging and quantitative analysis of atherosclerotic lesions by CARS-based multimodal nonlinear optical microscopy. Arterioscler. Thromb. Vasc. Biol..

[42] Zoumi A., Yeh A., Tromberg B.J. (2002). Imaging cells and extracellular matrix in vivo by using second-harmonic generation and two-photon excited fluorescence. Proc. Natl. Acad. Sci. USA.

[43] Lu P., Takai K., Weaver V.M., Werb Z. (2011). Extracellular matrix degradation and remodeling in development and disease. Cold Spring Harb. Perspect. Biol..

[44] Mecham R.P. (2012). Overview of extracellular matrix. Curr. Protoc. Cell Biol..

[45] Mouw J.K., Ou G., Weaver V.M. (2014). Extracellular matrix assembly: A multiscale deconstruction. Nat. Rev. Mol. Cell Biol..

[46] Rozario T., DeSimone D.W. (2010). The extracellular matrix in development and morphogenesis: A dynamic view. Dev. Biol..

[47] Barczyk M.M., Olsen L.H., da Franca P., Loos B.G., Mustafa K., Gullberg D., Bolstad A.I. (2009). A role for α11β1 integrin in the human periodontal ligament. J. Dent. Res..

[48] Dzamba B.J., Keene D.R., Isogai Z., Charbonneau N.L., Karaman-Jurukovska N., Simon M., Sakai L.Y. (2001). Assembly of epithelial cell fibrillins. J. Investig. Dermatol..

[49] Ramirez F., Sakai L.Y. (2010). Biogenesis and function of fibrillin assemblies. Cell Tissue Res..

[50] Durbeej M. (2010). Laminins. Cell Tissue Res..

[51] Gordon M.K., Hahn R.A. (2010). Collagens. Cell Tissue Res..

[52] Brodsky B., Persikov A.V. (2005). Molecular structure of the collagen triple helix. Adv. Protein Chem..

[53] Celerin M., Ray J.M., Schisler N.J., Day A.W., Stetler-Stevenson W.G., Laudenbach D.E. (1996). Fungal fimbriae are composed of collagen. EMBO J..

[54] King N., Westbrook M.J., Young S.L., Kuo A., Abedin M., Chapman J., Fairclough S., Hellsten U., Isogai Y., Letunic I. (2008). The genome of the choanoflagellate Monosiga brevicollis and the origin of metazoans. Nature.

[55] Rasmussen M., Jacobsson M., Bjorck L. (2003). Genome-based identification and analysis of collagen-related structural motifs in bacterial and viral proteins. J. Biol. Chem..

[56] Fratzl P., Misof K., Zizak I., Rapp G., Amenitsch H., Bernstorff S. (1998). Fibrillar structure and mechanical properties of collagen. J. Struct. Biol..

[57] Hulmes D.J. (2002). Building collagen molecules, fibrils, and suprafibrillar structures. J. Struct. Biol..

[58] Hulmes D.J. (2008). Collagen Structure and Mechanics.

[59] Myllyharju J., Kivirikko K.I. (2004). Collagens, modifying enzymes and their mutations in humans, flies and worms. Trends Genet..

[60] Ricard-Blum S., Ruggiero F. (2005). The collagen superfamily: From the extracellular matrix to the cell membrane. Pathol. Biol..

[61] Cicchi R., Vogler N., Kapsokalyvas D., Dietzek B., Popp J., Pavone F.S. (2013). From molecular structure to tissue architecture: Collagen organization probed by SHG microscopy. J. Biophotonics.

[62] Dunn K.W., Young P.A. (2006). Principles of multiphoton microscopy. Nephron Exp. Nephrol..

[63] Xu C., Zipfel W., Shear J.B., Williams R.M., Webb W.W. (1996). Multiphoton fluorescence excitation: New spectral windows for biological nonlinear microscopy. Proc. Natl. Acad. Sci. USA.

[64] Bass M., van Strvland E.W., Williams D.R., Wolfe W.L. (2001). Handbook of Optics.

[65] Sutherland R.L. (2003). Handbook of Nonlinear Optics.

[66] Svirko Y.P., Zheludev N.I. (2000). Polarization of Light in Nonlineear Optics.

[67] Boyd G.D., Kleinman D.A. (1968). Parametric interation of focused Gaussian light beams. J. Appl. Phys..

[68] Brjorkholm J.E. (1966). Opitcal second-harmonic generation using a focused Gaussian laser beam. Phys. Rev..

[69] Dmitriev V.G., Gurzadyan G.G., Nikogosyan D.N., Lotsch H.K.V. (1999). Handbook of Nonlinear Optical Crystals.

[70] Sapaev U.K., Kulagin I.A., Usmanov T. (2003). Theory of second-harmonic generation for limited laser beams in nonlinear crystals. J. Opt. B Quantum Semiclass. Opt..

[71] Chen X., Nadiarynkh O., Plotnikov S., Campagnola P.J. (2012). Second harmonic generation microscopy for quantitative analysis of collagen fibrillar structure. Nat. Protoc..

[72] Masters B.R., So P. (2008). Handbook of Biomedical Nonlinear Optical Microscopy.

[73] Roth S., Freund I. (1979). Second harmonic generation in collagen. J. Chem. Phys..

[74] Theodossiou T.A., Thrasivoulou C., Ekwobi C., Becker D.L. (2006). Second harmonic generation confocal microscopy of collagen type I from rat tendon cryosections. Biophys. J..

[75] Stoller P., Kim B.M., Rubenchik A.M., Reiser K.M., Da Silva L.B. (2002). Polarization-dependent optical second-harmonic imaging of a rat-tail tendon. J. Biomed. Opt..

[76] Helmchen F., Fee M.S., Tank D.W., Denk W. (2001). A miniature head-mounted two-photon microscope: High-resolution brain imaging in freely moving animals. Neuron.

[77] Llewellyn M.E., Barretto R.P., Delp S.L., Schnitzer M.J. (2008). Minimally invasive high-speed imaging of sarcomere contractile dynamics in mice and humans. Nature.

[78] Wu Y., Leng Y., Xi J., Li X. (2009). Scanning all-fibre-optic endomicroscopy system for 3D nonlinear optical imaging of biological tissues. Opt. Express.

[79] Crisafi F., Kumar V., Perri A., Marangoni M., Cerullo G., Polli D. (2017). Multimodal nonlinear microscope based on a compact fibre-format laser source. Spectrochim. Acta A Mol. Biomol. Spectrosc..

[80] Song W., Xu Q., Zhang Y., Zhan Y., Zheng W., Song L. (2016). Fully integrated reflection-mode photoacoustic, two-photon, and second harmonic generation microscopy in vivo. Sci. Rep..

[81] Atsuta K., Ogura Y., Hase E., Minamikawa T., Yasui T. (2017). In situ monitoring of collagen fibres in human skin using a photonic-crystal-fibre-coupled, hand-held, second-harmonic-generation microscope. Proc. SPIE.

[82] Sanchez G.N., Sinha S., Liske H., Chen X., Nguyen V., Delp S.L., Schnitzer M.J. (2015). In Vivo Imaging of Human Sarcomere Twitch Dynamics in Individual Motor Units. Neuron.

[83] Williams J.C., Campagnola P.J. (2015). Wearable Second Harmonic Generation Imaging: The Sarcomeric Bridge to the Clinic. Neuron.

[84] Chen G., Chen J., Zhuo S., Xiong S., Zeng H., Jiang X., Chen R., Xie S. (2009). Nonlinear spectral imaging of human hypertrophic scar based on two-photon excited fluorescence and second-harmonic generation. Br. J. Dermatol..

[85] Rosin N.L., Agabalyan N., Olsen K., Martufi G., Gabriel V., Biernaskie J., Di Martino E.S. (2016). Collagen structural alterations contribute to stiffening of tissue after split-thickness skin grafting. Wound Repair Regen..

[86] Su P.J., Chen W.L., Hong J.B., Li T.H., Wu R.J., Chou C.K., Chen S.J., Hu C., Lin S.J., Dong C.Y. (2009). Discrimination of collagen in normal and pathological skin dermis through second-order susceptibility microscopy. Opt. Express.

[87] Liu S.H., Yang R.S., al-Shaikh R., Lane J.M. (1995). Collagen in tendon, ligament, and bone healing. A current review. Clin. Orthop. Relat. Res..

[88] Mansfield J.C., Winlove C.P., Moger J., Matcher S.J. (2008). Collagen fibre arrangement in normal and diseased cartilage studied by polarization sensitive nonlinear microscopy. J. Biomed. Opt..

[89] Sivaguru M., Durgam S., Ambekar R., Luedtke D., Fried G., Stewart A., Toussaint K.C. (2010). Quantitative analysis of collagen fibre organization in injured tendons using Fourier transform-second harmonic generation imaging. Opt. Express.

[90] Doras C., Taupier G., Barsella A., Mager L., Boeglin A., Bulou H., Bousquet P., Dorkenoo K.D. (2011). Polarization state studies in second harmonic generation signals to trace atherosclerosis lesions. Opt. Express.

[91] Lilledahl M.B., Haugen O.A., de Lange Davies C., Svaasand L.O. (2007). Characterization of vulnerable plaques by multiphoton microscopy. J. Biomed. Opt..

[92] Megens R.T., oude Egbrink M.G., Merkx M., Slaaf D.W., van Zandvoort M.A. (2008). Two-photon microscopy on vital carotid arteries: Imaging the relationship between collagen and inflammatory cells in atherosclerotic plaques. J. Biomed. Opt..

[93] Mostaço-Guidolin L.B., Sowa M.G., Ridsdale A., Pegoraro A.F., Smith M.S., Hewko M.D., Kohlenberg E.K., Schattka B., Shiomi M., Stolow A. (2010). Differentiating atherosclerotic plaque burden in arterial tissues using femtosecond CARS-based multimodal nonlinear optical imaging. Biomed. Opt. Express.

[94] Abraham T., Fong G., Scott A. (2011). Second harmonic generation analysis of early Achilles tendinosis in response to in vivo mechanical loading. BMC Musculoskelet. Disord..

[95] Kottmann R.M., Sharp J., Owens K., Salzman P., Xiao G.Q., Phipps R.P., Sime P.J., Brown E.B., Perry S.W. (2015). Second harmonic generation microscopy reveals altered collagen microstructure in usual interstitial pneumonia versus healthy lung. Respir. Res..

[96] Tjin G., Xu P., Kable S.H., Kable E.P., Burgess J.K. (2014). Quantification of collagen I in airway tissues using second harmonic generation. J. Biomed. Opt..

[97] Tan H.Y., Sun Y., Lo W., Teng S.W., Wu R.J., Jee S.H., Lin W.C., Hsiao C.H., Lin H.C., Chen Y.F. (2007). Multiphoton fluorescence and second harmonic generation microscopy for imaging infectious keratitis. J. Biomed. Opt..

[98] Lutz V., Sattler M., Gallinat S., Wenck H., Poertner R., Fischer F. (2012). Impact of collagen crosslinking on the second harmonic generation signal and the fluorescence lifetime of collagen autofluorescence. Skin Res. Technol..

[99] Pena A.M., Fagot D., Olive C., Michelet J.F., Galey J.B., Leroy F., Beaurepaire E., Martin J.L., Colonna A., Schanne-Klein M.C. (2010). Multiphoton microscopy of engineered dermal substitutes: Assessment of 3-D collagen matrix remodeling induced by fibroblast contraction. J. Biomed. Opt..

[100] Wolf K.A.S., Schacht V., Coussens L.M., von Andrian U.H., van Rheenen J., Deryugina E., Friedl P. (2009). Collagen-based cell migration models in vitro and in vivo. Semin. Cell Dev. Biol..

[101] Gurtner G.C., Werner S., Barrandon Y., Longaker M.T. (2008). Wound repair and regeneration. Nature.

[102] Rahmani W., Abbasi S., Hagner A., Raharjo E., Kumar R., Hotta A., Magness S., Metzger D., Biernaskie J. (2014). Hair follicle dermal stem cells regenerate the dermal sheath, repopulate the dermal papilla, and modulate hair type. Dev. Cell.

[103] Liu Y., Zhu X., Huang Z., Cai J., Chen R., Xiong S., Chen G., Zeng H. (2015). Texture analysis of collagen second-harmonic generation images based on local difference local binary pattern and wavelets differentiates human skin abnormal scars from normal scars. J. Biomed. Opt..

[104] Junker J.P., Philip J., Kiwanuka E., Hackl F., Caterson E.J., Eriksson E. (2014). Assessing quality of healing in skin: Review of available methods and devices. Wound Repair Regen..

[105] Kumar N., Kumar P., Nayak Badagabettu S., Prasad K., Kudva R., Vasudevarao R.C. (2014). Surgical implications of asymmetric distribution of dermal collagen and elastic fibres in two orientations of skin samples from extremities. Plast. Surg. Int..

[106] Verhaegen P.D., van Zuijlen P.P., Pennings N.M., van Marle J., Niessen F.B., van der Horst C.M., Middelkoop E. (2009). Differences in collagen architecture between keloid, hypertrophic scar, normotrophic scar, and normal skin: An objective histopathological analysis. Wound Repair Regen..

[107] Hollinsky C., Sandberg S. (2007). Measurement of the tensile strength of the ventral abdominal wall in comparison with scar tissue. Clin. Biomech..

[108] Bateman E.D., Turner-Warwick M., Adelmann-Grill B.C. (1981). Immunohistochemical study of collagen types in human foetal lung and fibrotic lung disease. Thorax.

[109] Thibeault D.W., Mabry S.M., Ekekezie I.I., Zhang X., Truog W.E. (2003). Collagen scaffolding during development and its deformation with chronic lung disease. Pediatrics.

[110] Bradley K.H., McConnell S.D., Crystal R.G. (1974). Lung collagen composition and synthesis. Characterization and changes with age. J. Biol. Chem..

[111] Mizikova I., Ruiz-Camp J., Steenbock H., Madurga A., Vadasz I., Herold S., Mayer K., Seeger W., Brinckmann J., Morty R.E. (2015). Collagen and elastin cross-linking is altered during aberrant late lung development associated with hyperoxia. Am. J. Physiol. Lung Cell. Mol. Physiol..

[112] Pena A.M., Fabre A., Debarre D., Marchal-Somme J., Crestani B., Martin J.L., Beaurepaire E., Schanne-Klein M.C. (2007). Three-dimensional investigation and scoring of extracellular matrix remodeling during lung fibrosis using multiphoton microscopy. Microsc. Res. Tech..

[113] Raub C.B., Mahon S., Narula N., Tromberg B.J., Brenner M., George S.C. (2010). Linking optics and mechanics in an in vivo model of airway fibrosis and epithelial injury. J. Biomed. Opt..

[114] Abraham T., Hirota J.A., Wadsworth S., Knight D.A. (2011). Minimally invasive multiphoton and harmonic generation imaging of extracellular matrix structures in lung airway and related diseases. Pulm. Pharmacol. Ther..

[115] Abraham T., Hogg J. (2010). Extracellular matrix remodeling of lung alveolar walls in three dimensional space identified using second harmonic generation and multiphoton excitation fluorescence. J. Struct. Biol..

[116] Hackett T.L., Ferrante S.C., Hoptay C.E., Engelhardt J.F., Ingram J.L., Zhang Y., Alcala S.E., Shaheen F., Matz E., Pillai D.K. (2017). A Heterotopic Xenograft Model of Human Airways for Investigating Fibrosis in Asthma. Am. J. Respir. Cell Mol. Biol..

[117] Campbell J.D., McDonough J.E., Zeskind J.E., Hackett T.L., Pechkovsky D.V., Brandsma C.A., Suzuki M., Gosselink J.V., Liu G., Alekseyev Y.O. (2012). A gene expression signature of emphysema-related lung destruction and its reversal by the tripeptide GHK. Genome Med..

[118] Mostaço-Guidolin L.B., Osei E.T., Hajimohammadi S., Ullah J., Hackett T.-L. (2016). Novel non-linear optical imaging to understand the composition of firbrilar collagen and elastin in remodeled asthmatic airways. Am. J. Respir. Crit. Care Med..

[119] Khan M.G. (2005). Encyclopedia of Heart Disease.

[120] Furchgott R.F. (1983). Role of endothelium in responses of vascular smooth muscle. Circ. Res..

[121] Ross R. (1999). Atherosclerosis is an inflammatory disease. Am. Heart J..

[122] Chen X., Huang Z., Xi G., Chen Y., Lin D., Wang J., Li Z., Sun L., Chen J., Chen R. (2011). Quantitative analysis of collagen change between normal and cancerous thyroid tissues based on SHG method. Proc. SPIE.

[123] Han X., Burke R.M., Zettel M.L., Tang P., Brown E.B. (2008). Second harmonic properties of tumor collagen: Determining the structural relationship between reactive stroma and healthy stroma. Opt. Express.

[124] Boulesteix T., Pena A.M., Pages N., Godeau G., Sauviat M.P., Beaurepaire E., Schanne-Klein M.C. (2006). Micrometer scale ex vivo multiphoton imaging of unstained arterial wall structure. Cytometry A.

[125] Van Zandvoort M., Engels W., Douma K., Beckers L., Oude Egbrink M., Daemen M., Slaaf D.W. (2004). Two-photon microscopy for imaging of the (atherosclerotic) vascular wall: A proof of concept study. J. Vasc. Res..

[126] Fratzl P. (2008). Collagen: Structure and Mechanics.

[127] Ottani V., Raspanti M., Ruggeri A. (2001). Collagen structure and functional implications. Micron.

[128] Heybeli N.K.B., Yilmaz B., Guler O. (2016). Musculoskeletal Research and Basic Science—“Tendons and ligaments”.

[129] Kannus P. (2000). Structure of the tendon connective tissue. Scand. J. Med. Sci. Sports.

[130] Keene D.R., Sakai L.Y., Bachinger H.P., Burgeson R.E. (1987). Type III collagen can be present on banded collagen fibrils regardless of fibril diameter. J. Cell Biol..

[131] Shimomura T., Jia F., Niyibizi C., Woo S.L. (2003). Antisense oligonucleotides reduce synthesis of procollagen α1 (V) chain in human patellar tendon fibroblasts: Potential application in healing ligaments and tendons. Connect. Tissue Res..

[132] Sivaguru M.D.S., Ambekar R., Luedtke D., Fried G., Stewart A., Toussaint K.C. (2011). Quantitative analysis of diseased horse tendons using Fourier-tranform-second-harmonic generation imaging. Proc. SPIE.

[133] Malik R.A., Kallinikos P., Abbott C.A., van Schie C.H., Morgan P., Efron N., Boulton A.J. (2003). Corneal confocal microscopy: A non-invasive surrogate of nerve fibre damage and repair in diabetic patients. Diabetologia.

[134] Raub C.B., Putnam A.J., Tromberg B.J., George S.C. (2010). Predicting bulk mechanical properties of cellularized collagen gels using multiphoton microscopy. Acta Biomater..

[135] Kirkpatrick N.D., Hoying J.B., Botting S.K., Weiss J.A., Utzinger U. (2006). In vitro model for endogenous optical signatures of collagen. J. Biomed. Opt..

[136] Turcotte R., Mattson J.M., Wu J.W., Zhang Y., Lin C.P. (2016). Molecular Order of Arterial Collagen Using Circular Polarization Second-Harmonic Generation Imaging. Biophys. J..

[137] Rao R.A., Mehta M.R., Leithem S., Toussaint K.C. (2009). Quantitative analysis of forward and backward second-harmonic images of collagen fibres using Fourier transform second-harmonic-generation microscopy. Opt. Lett..

[138] Williams R.M., Zipfel W.R., Webb W.W. (2005). Interpreting second-harmonic generation images of collagen I fibrils. Biophys. J..

[139] Indahl U., Næs T. (1998). Evaluation of alternative spectral feature extraction methods of textural images for multivariate modeling. J. Chemom..

[140] Chang T., Kuo C.J. (1993). Texture analysis and classification with tree-structured wavelet transform. IEEE Trans Image Process.

[141] Tuceryan M.J., Jain A.K., Chen C.H. (1993). Texture Analysis. Handbook of Pattern Recognition and Comuter Vision.

[142] Unser M. (1995). Texture classification and segmentation using wavelet frames. IEEE Trans. Image Process..

[143] Arivazhagan S.G.L. (2003). Texture classfication using wavelet transform. Pattern Recognit. Lett..

[144] Mostaço-Guidolin L.B., Ko A.C., Popescu D.P., Smith M.S., Kohlenberg E.K., Shiomi M., Major A., Sowa M.G. (2011). Evaluation of texture parameters for the quantitative description of multimodal nonlinear optical images from atherosclerotic rabbit arteries. Phys. Med. Biol..

[145] Bharati M.H., Liu J.J., MacGregor J.F. (2004). Image texture analysis: Methods and comparisons. Chemom. Intell. Lab. Syst..

